# Central venous catheter infection-related glomerulonephritis under long-term parenteral nutrition: a report of two cases

**DOI:** 10.1186/s13104-016-1997-3

**Published:** 2016-03-31

**Authors:** Mari Okada, Mai Sato, Masao Ogura, Koichi Kamei, Kentaro Matsuoka, Shuichi Ito

**Affiliations:** Division of Nephrology and Rheumatology, National Center for Child Health and Development, Tokyo, Japan; Department of Pediatrics, Musashino Red Cross Hospital, 1-26-1 Kyonancho, Musashino, Tokyo, 180-8610 Japan; Division of Pathology, National Center for Child Health and Development, Tokyo, Japan; Department of Pediatrics, Graduate School of Medicine, Yokohama City University, 3-9 Fukuura, Kanazawaku, Yokohama, 236-004 Japan

**Keywords:** Central venous catheters, Membranoproliferative glomerulonephritis, *Staphylococcus epidermidis*, Anti-neutrophil cytoplasmic antibodies, Shunt nephritis, Megacystis microcolon intestinal hypoperistalsis syndrome

## Abstract

**Background:**

Advances in long-term parenteral nutrition via indwelling central venous catheter have improved the quality of life and mortality in patients with life-threatening gastrointestinal diseases complicated with severely impaired absorption. However, infection to central venous catheter is still a common and critical complication for such patients. We encountered two patients under long-term parenteral nutrition who developed glomerulonephritis associated with central venous catheter infection. Persistent bacterial infection in indwelling medical devices placed in the blood-stream such as a ventricular-atrial shunt is known to cause glomerulonephritis, a condition termed shunt nephritis. We reported the clinical manifestations, treatment and their pathological findings in the two patients with glomerulonephritis associated with central venous catheter infection.

**Case presentation:**

Both patients suffered from megacystis microcolon intestinal hypoperistalsis syndrome, a form of pseudo-Hirschsprung’s disease. They had been receiving home parenteral nutrition via central venous catheter because of severe malabsorption. They presented proteinuria, hematuria, hypocomplementemia and positive PR3-antineutrophilic cytoplasmic antibody accompanied by *Staphylococcus epidermidis* infection in the central venous catheter. Their renal biopsy revealed membranoproliferative glomerulonephritis with positive C3 deposition. One of them recovered completely following the removal of catheter and administration of antibiotics, while another did not respond to the treatments. We then treated her with methylprednisolone pulse therapy followed by prednisolone. She responded well, and achieved complete remission.

**Conclusion:**

As central venous catheter infection-related glomerulonephritis has a similar etiology to shunt nephritis, removal of the catheter and administration of antibiotics is fundamental to the treatment. If a patient is resistant to such conventional therapy, additional steroid and/or immunosuppressive agent could be considered. Although the number of patients with classical shunt nephritis is decreasing since the ventricular-peritoneal shunt has become became the major procedure for hydrocephalus, central venous catheter infection-related glomerulonephritis may increase in the future due to a marked increase in the number of patients receiving long-term parenteral nutrition. Routine urinalysis should be considered in such patients for early detection of central venous catheter infection-related glomerulonephritis.

## Background

Advanced in total parenteral nutrition via central venous catheter (CVC) have rescued many patients with congenital or acquired life-threatening gastrointestinal disease broad resection of intestine complicated with severe malabsorption, as well as conditions such as short bowel syndrome, inflammatory bowel diseases, intractable diarrhea and malignancy. In recent years, it is not uncommon for patients who are under end-of-life care often receive total parenteral nutrition resulting in a rising, number of patients receiving long-term home parenteral nutrition (HPN). The flip side of HPN, however, is bacterial or fungal infection in indwelling catheter. It is a critical complication that influences the patient’s mortality.

Glomerulonephritis associated with CVC-related infection is a rare complication of long-term total parenteral nutrition. Persistent bacterial infection in indwelling medical devices in blood-stream, such as ventricular-atrial shunt (V-A shunt), rarely causes immune-complex-mediated glomerulonephritis. A representative example is shunt nephritis, which occurs in approximately 0.7–2.3 % of patients with chronic V-A shunt infections mainly caused by *S. epidermidis*, and *coagulase*-*negative staphylococcus* [1]. Membranoproliferative glomerulonephritis with deposits of C3, IgM, and IgG is the most frequent renal pathological finding of shunt nephritis. The etiology of CVC infection-related glomerulonephritis may be similar to that of shunt nephritis. However, there have been few reports of CVC infection-related glomerulonephritis. Here, we describe our encounter with two patients who had glomerulonephritis associated with CVC infection.

## Case presentation

### Case 1

A 12-year-old boy was admitted to our hospital for further evaluation of proteinuria, and renal insufficiency. He had megacystis microcolon intestinal hypoperistalsis syndrome (MMIHS), a form of pseudo-Hirschsprung’s disease. He had been on HPN by CVC for 8 years. Two months before admission, he presented with macroscopic hematuria. He developed proteinuria and renal insufficiency 1 month before admission. On admission, his height was 137.8 cm (−1.49 SD) and body weight was 34.2 kg (−0.83 SD). His blood pressure was 100/54 mmHg, and the chest and abdomen exhibited no abnormal findings. Blood examination revealed hypoalbuminemia (serum albumin of 3.1 g/dL), renal insufficiency (serum creatinine of 0.68 mg/dL), estimated glomerular filtration rate (e-GFR), 70.8 mL/min/1.73 m^2^, hypocomplementemia (C3, 4 mg/dL, C4, 3.4 mg/dL, and CH50, 10 U/mL) and positive PR3-anti-neutrophil cytoplasmic antibodies (ANCA) (33 U/mL; normal range 0–9 U/mL). Urinalysis revealed proteinuria (urinary protein 137.0 mg/dL, urinary creatinine, 63.5 mg/dL), and hematuria (>100 erythrocytes per high power field). Renal biopsy was performed after admission. Light microscopy (LM) revealed mesangial proliferation with one crescent formation in 23–30 glomeruli. Immunofluorescence microscopy revealed C3, C1q and immunoglobulin (Ig) M deposits along the capillary, and in mesangial region. Electron microscopy (EM) revealed paramesangial deposits. The pathological findings were consistent with membranoproliferative glomerulonephritis. We treated him with 60 mg of oral prednisolone and 150 mg of mizoribine for 10 days, but his urinary findings and renal function did not improve. He suddenly developed fever on the 12th day after admission, and blood culture did not reveal *S. epidermidis* (MSSE) colonization in the peripheral blood but in the CVC instead. We immediately stopped both prednisolone and mizoribine, removed the CVC, and administered cefazolin for 10 days.

After the removal of CVC, his renal function gradually improved, and proteinuria and hematuria spontaneously disappeared in 6 months (Fig. 2a). Recurrence of proteinuria and hematuria has not been occurred.

### Case 2

A 24-year-old woman was admitted to our hospital due to fever, hematuria, proteinuria, and renal insufficiency. She had suffered from MMIHS, and had been on HPN by CVC for 18 years. Three weeks before admission, she presented with fever. Two weeks before admission, her urine output started to decrease. She developed edema in her lower extremities and gained 3 kg in weight.

**Fig. 1:**
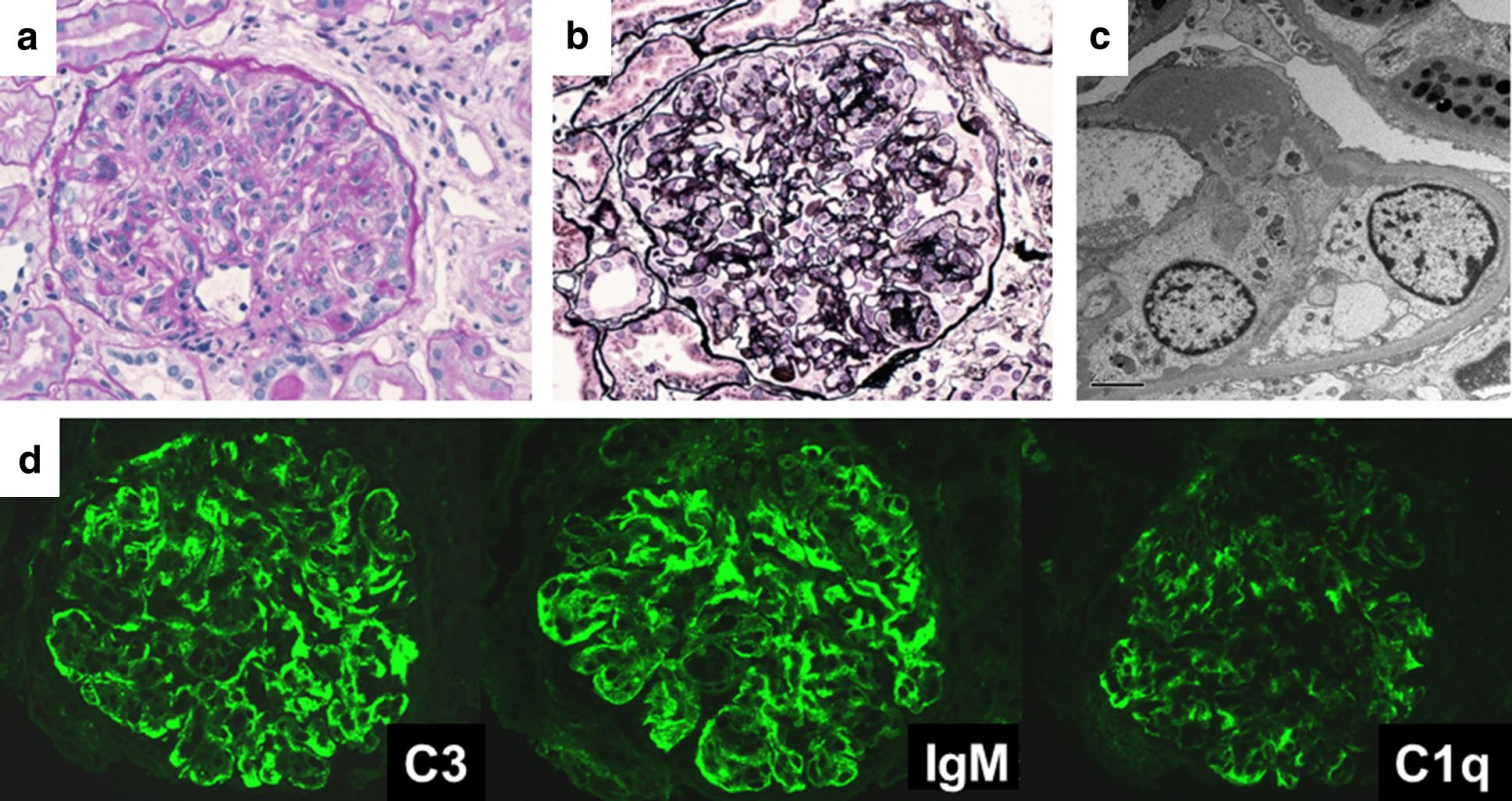
First biopsy of patient 2: **a** (PAS stain, magnification ×400); **b** (PAM stain, magnification ×400), mesangial proliferation, increased lobulation, doubled contours: **c** (electron microscopy), subepithelial, subendothelial, mesangial deposit: **d** (immuno-fluorescence microscopy), C3 (++), IgM (++), C1q (+), IgG (+) fringe pattern

**Fig. 2:**
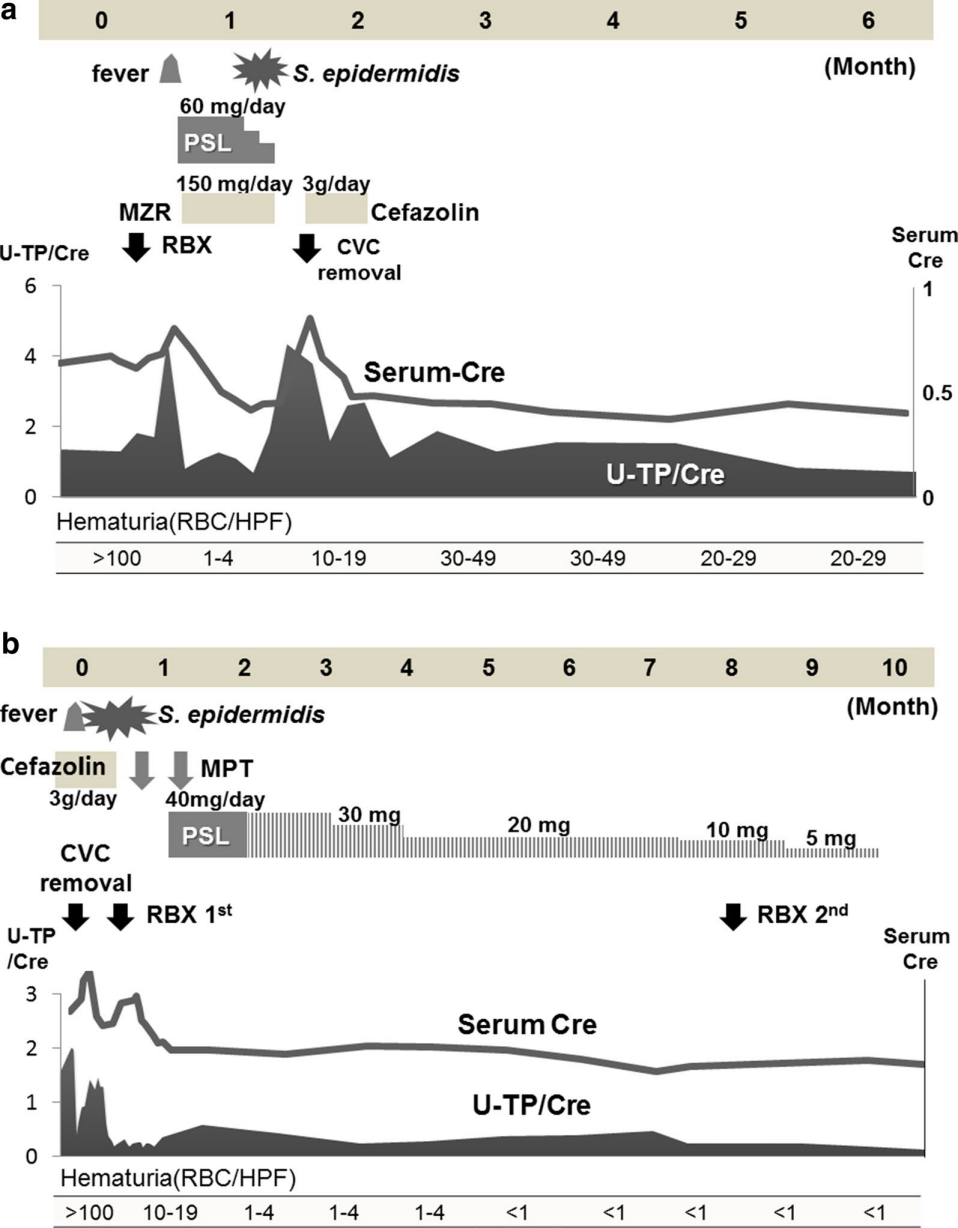
**a** Clinical course of patient 1, **b** clinical course of patient 2

On admission, her height was 159.4 cm, and body weight was 45.8 kg. Her blood pressure was 110/82 mmHg, and her chest and abdomen exhibited no abnormal findings. Blood examination revealed hypoalbuminemia (serum albumin, 2.5 g/dL), renal insufficiency (serum creatinine, 0.92 mg/dL, Cys-C 1.69 mg/L, e-GFR, 63.1 mL/min/1.73 m^2^, hypocomplementemia (C3, 57 mg/dL; C4, 24 mg/dL; and CH50, 10.5 U/mL) and positive PR3-ANCA (19 U/mL; normal range 0-9 U/mL). Urinalysis revealed proteinuria (urinary protein 29.1 mg/dL, urinary creatinine 18.7 mg/dL), and hematuria (30–49 erythrocytes per high power field). Blood cultures did not reveal *S. epidermidis* (MRSE) colonization in the peripheral blood but in the CVC instead. After admission, we immediately removed the CVC and started antibiotics for 7 days. Her fever was soon resolved, but proteinuria, hematuria and renal insufficiency continued. Renal biopsy was performed 18 days after admission (Fig. 1). LM revealed diffuse mesangial proliferation, lobulation in the glomeruli, endocapillary proliferation and double contours in the glomerular capillary. Two of 18 glomeruli showed global glomerular sclerosis. Immunofluorescence microscopy revealed deposits of C3 and IgM with fringe pattern along the glomerular capillary. EM revealed subepithelial, subendothelial, and intra-basement membrane electron-dense deposits. These biopsy findings are compatible with membranoproliferative glomerulonephritis. After several blood cultures that proved to be negative, she was treated with two courses of methylprednisolone pulse therapy followed by 40 mg prednisolone daily for 1 month. Afterwards, prednisolone was reduced to 40 mg every other day and gradually decreased over 8 months. She responded well to these treatments, urinary findings and renal function completely recovered in 9 months (Fig. 2b). She remains free from medication without further recurrence of proteinuria and hematuria.

## Conclusion

CVC infection-related glomerulonephritis is an immune complex-mediated disease accompanied by persistent infection of *S. epidermidis*. Its etiology may be the same as that of shunt nephritis.

Shunt nephritis was first reported in 1965 by Black et al. [2] who described two children with nephrotic syndrome associated with infection by coagulase-negative staphylococcus in the V-A shunt. Shunt nephritis is often accompanied by hypocomplementemia (89–90 % of cases), positive PR3-ANCA, renal insufficiency (50 %), and nephritic syndrome (30 %). *S. epidermidis* infection accounts for 75 % of all shunt nephritis cases [1, 3]. While over half of the individuals with shunt nephritis achieved complete recovery, 40 % developed persistent urine abnormalities or end-stage renal disease (ESRD), and 9 % experienced death [1]. Wyatt et al. [5] reported the serial complement profile in patients with shunt nephritis. A decrease in the serum levels of C1q, C3 and C4 due to activation of classical pathway occurred, but, was resolved after treatment. The two patients in our case showed compatible clinical manifestations and laboratory findings, suggesting a similar etiology. Previously, Kusaba et al. [5] had also speculated that the classical complement pathway might be involved in CVC infection-related glomerulonephritis because immunofluorescence microscopy revealed C3 and IgM deposits and hypocomplementemia disappeared after catheter removal.

Although, staphylococcus infection-associated glomerulonephritis and lupus nephritis are given in differential diagnosis, CVC infection-related glomerulonephritis revealed a different pattern of immunofluorescent staining. Furthermore, PR-3 ANCA often becomes positive while anti-dsDNA antibody is negative in CVC infection-related glomerulonephritis.

In the previous literatures, there were only four patients with CVC infection-related glomerulonephritis [5–7]. All of them had abnormal urinary findings, renal impairment and hypocomplementemia that improved after CV catheter removal, except one who developed ESRD. Among them, the details of two patients were not described [7]. Therefore, we have included the details of these two patients together with those of our patients in Table 1.

**Table 1:** CVC infection-related glomerulonephritis in published reports and our patients

	Ohara et al.	Kusaba et al.	Patient 1	Patient 2
Age, sex	13, M	59, F	12, M	24, F
Underlying disease	Short bowel syndrome	Post-radiation enteritis	MMIHS	MMIHS
Duration of CVC (years)	13	2	8	18
Blood culture	*S. epidermidis*	*S. epidermidis*	*S. epidermidis* (MSSE)	*S. epidermidis* (MRSE)
24 h CCr/e-GFR (mL/min/1.73 m^2^)	84.6/−	−/5.3	77.4/70.8	−/63.1
C3/C4/CH50	30/8/ <10	30/10/ <12	69/7.4/26.4	57/24/10.5
PR3-ANCA	N/D	<5	33	19
Renal pathology (positive IF)	MPGN C3, IgM, C1	Crescentic GN C3, IgM, IgG	MPGN C3, IgM, C1q	MPGN C3, IgM
Treatment	CVC removal	CVC removal	CVC removal	CVC removal
		Cefazolin for 10 days	Cefazolin (3 g/day) for 10 days	Cefazolin (3 g/day) for 7 days
			PSL 60 mg/day + MZR 150 mg/day for 10 days	MPT 2 courses + PSL 40 mg/day for 1 month
Outcome	Full recovery	ESRD	Full recovery	Full recovery

*MMIHS* megacystis microcolon intestinal hypoperistasis syndrome, *CVC* central venous catheter, *CCr* creatinine clearance, *e-GFR* estimated glomerular filtration rate, *GN* glomerulonephritis, *MPGN* membranoproliferative glomerulonephritis, *MPT* methyl prednisolone pulse therapy, *PSL* prednisolone, *ESRD* end stage renal disease

Our patients showed positive PR3-ANCA. Although the underlying mechanism for positive PR3-ANCA is unclear, a likely explanation is that when pathogens destroy neutrophils, PR-3 antigen may become exposed on the surface and results in the production of PR3-ANCA. Indeed, PR3-ANCA is positive not only in patients with vasculitis, but also in those with infections such as *Streptococcus* infection [8] and HIV [9]. PR3-ANCA was transiently positive in 33 % of patients with septic shock [10]. In our cases, PR3-ANCA became negative after the resolution of glomerulonephritis.

Most patients with CVC infection-related glomerulonephritis responded well to catheter removal and administration of antibiotics. However, Kusaba et al. [5] reported a 59-year-old woman with glomerulonephritis developed ESRD because catheter removal was delayed. The timing of CVC removal is difficult. The Clinical Practice Guidelines for the Diagnosis and Management of Intravascular Catheter-Related Infection 2009 by the Infectious Diseases Society of America proposed that in principal the treatment of CVC infection due to coagulase-negative staphylococci is CVC removal and administration of antibiotics for 5–7 days. However, when CVC removal is not possible, antibiotics administration and antibiotic lock therapy should be continued for 10–14 days. In the patient reported by Kusaba et al., inflammatory parameters improved after administration of antibiotics. However, hypocomplementemia persisted until removal of the CVC, with ESRD eventually developing. This suggested that administration of antibiotics alone was insufficient. Therefore, early detection, prompt catheter removal and treatment with antibiotics are essential the same treatment strategy against shunt nephritis. Although patient 2 did not respond to catheter removal and administration of antibiotics, steroid therapy was effective. In spite of Patient 2 having MRSE colonization, we administered cefazoline for 7 days. While it might contribute to continuous proteinuria, hematuria, and renal insufficiency, it was unlikely that catheter-related blood stream infection continued because the patient’s fever dropped immediately and blood culture was negative after the removal of the CVC. Steroids should be administered carefully to patients who have infectious disease. We treated patient 1 with steroids and mizoribine before administration of antibiotics because we did not notice his catheter infection until he developed fever. Therefore, we stopped administration of steroids and mizoribine when blood culture revealed MSSE colonization. In the event that several sets of blood cultures proved negative after the removal of infected catheter, additional treatment with steroid and/or immunosuppressive agent can be considered based on the pathogenesis. Similarly, efficacy of steroid against refractory shunt nephritis after replacement of the V-A shunt has been reported [11]. At present, the dose of prednisolone is not fixed now. Iwata et al. [11] initiated oral prednisolone at a dose of 50 mg/day after replacement of shunt. In our case, we used a dose of prednisolone based on the guideline for IgA nephritis.

There is a possibility that the severity of underlying disease is involved in the outcome. To date, however there are have been no reports of patients diagnosed with renal insufficiency before CVC infection in previous reports. Therefore, early detection and treatment are the most crucial to improve prognosis.

Although the numbers of patients with shunt nephritis is decreasing because ventricular-peritoneal shunt has become the major procedure for hydrocephalus (instead of V-A shunt), cases of CVC infection-related glomerulonephritis may increase because more patients will be treated with long-term parenteral nutrition. Meanwhile, some patients with CVC infection-related glomerulonephritis may be overlooked because of mild or chronic manifestation or spontaneous recovery by removal of the CVC at the time of the infection. However, to prevent a delay in detection, routine urinalysis is recommended in patients receiving long-term parenteral nutrition via indwelling CVC.

## Consent

Written informed consent was obtained from the patient or patient’s parents for publication of this case report and any accompanying images.

## Abbreviations

CVC: central venous catheter
HPN: home parenteral nutrition
VA-shunt: ventricular-atrial shunt
MMIHS: megacystis microcolon intestinal hypoperistalsis syndrome
LM: light microscopy
EM: electron microscopy
ANCA: anti-neutrophil cytoplasmic antibodies
ESRD: end stage renal disease
